# Capsaicin suppresses liver fat accumulation in high-fat diet-induced NAFLD mice

**DOI:** 10.1080/19768354.2020.1810771

**Published:** 2020-08-26

**Authors:** Mi Kyung Shin, Soo-Man Yang, In-Seob Han

**Affiliations:** a Department of Biological Sciences, University of Ulsan, Ulsan, Korea; b Department of Pathology, Hanyang University, Seongdong-gu, Seoul, Korea

**Keywords:** Capsaicin, high-fat diet (HFD), NAFLD, fatty acid, metabolism

## Abstract

Dietary capsaicin exhibits anti-steatosis activity in obese mice. High-fat diet (HFD)-induced mice is a highly studied approach to develop non-alcoholic fatty liver disease (NAFLD). In this study, we determined whether the topical application of capsaicin can improve lesions of NAFLD. The HFD-induced mice were treated with daily topical application of capsaicin for 8 weeks. Topical application of capsaicin reduced liver fat in HFD-fed mice. Capsaicin stimulated carnitine palmitoyl transferase (CPT)-1 and CD36 expression, which are associated with β-oxidation and fatty acids influx of liver while it decreased the expression of key enzymes involved in the synthesis of fatty acids, such as acetyl Co-A carboxylase (ACC) and fatty acid synthase (FAS). Immunohistochemical analysis revealed the elevated level of adiponectin in liver tissue of the capsaicin-treated mice. These results suggest that the topical application of capsaicin suppresses liver fat accumulation through the upregulation of β-oxidation and *de novo* lipogenesis in HFD-induced NAFLD mice.

## Introduction

Steatosis (fatty liver) is an accumulation of fat in the liver. When this progresses to become associated with inflammation, it is known as steatohepatitis. Fatty liver disease is divided into alcohol-related and NAFLD. The prevalence of NAFLD, a frequent chronic liver condition has dramatically increased over the past 15 years. NAFLD may develop as a consequence of multiple processes (Polyzos et al. 2010) and is closely associated with two major epidemics, obesity and type 2 diabetes mellitus (Hamaguchi et al. 2012). The histologic spectrum of NAFLD ranges from fatty liver to non-alcoholic steatohepatitis (NASH) or even cirrhosis. While hepatic lipid accumulation may be derived from dietary intake, esterification of plasma free fatty acids (FFA), or hepatic de novo lipogenesis, a central abnormality in NAFLD is enhanced de novo lipogenesis (Softic et al. 2016). An analysis of obese patients with NAFLD found that while 60% of liver triglycerides are derived from plasma free fatty acid, which themselves are derived from adipose tissue lipolysis, 26% of liver fat in this individual came from lipogenesis and another 15% from dietary fat (Donnelly et al. 2005). NAFLD is strongly associated with obesity, which induces adipokine secretion, endoplasmic reticulum and oxidative stress at the cellular level (Finelli and Tarantino 2013).

Capsaicin is the major pungent ingredient in chili peppers and is a highly selective agonist for the transient receptor potential vanilloid 1 (TRPV1) channels. Several *in vitro* and pre-clinical studies proved the efficacy of capsaicin in attenuating metabolic disorders through activation of TRPV1 (Panchal et al. 2018). A study with dietary capsaicin in fat-fed mice did indeed confirm that adipose expression of peroxisome proliferator-activated receptor (PPAR)-γ was increased in the treated mice, whereas gain in weight and visceral fat mass were blunted (Chen et al. 2015). Capsaicin-rich diets represent a beneficial intervention in populations at high risk for non-alcoholic fatty liver disease (NAFLD) (Hu et al. 2017). This beneficial effect of chronic dietary capsaicin intake on NAFLD is mediated by preventing fatty liver *in vivo* through TRPV1 activation (Li et al. 2013). TRPV1 activation by dietary capsaicin prevents NAFLD through PPAR-δ-dependent autophagy enhancement in mice. In addition, TRPV1 is highly expressed in liver tissues (Miao et al. 2008). Response of TRPV1 by capsaicin modulated AMP-activated protein kinase (AMPK), PPARα, uncoupling protein 1 (UCP1), and glucagon-like 1 (GLP-1) (Panchul et al. 2018).

In previous studies, we found that the topical application of capsaicin on the high-fat diet (HFD)-induced obese mice enhanced adiponectin level in visceral adipose tissues (Lee et al. 2013). Visceral obesity is the main risk factor for NAFLD, and inappropriate storage of triglycerides in adipocytes and higher concentrations of free fatty acids may add to increased hepatic lipid storage and progressive liver damage (Buechler et al. 2011). Capsaicin attenuated inflammation in liver tissue, which might be induced by phosphorylation of AMPK signaling through the TRPV1 receptor (Kang et al. 2011). This hepatic AMPK activation is mediated by systemic increases in the concentration of adiponectin as the mechanisms of action of capsaicin. Treatment of topical capsaicin with exercise ameliorated the symptoms of metabolic syndrome induced by hypoestrogenism by activating AMPK (Medina-Contreras et al. 2017). In the current study, we studied whether the topical application of capsaicin improves fatty liver through the inhibition of lipogenesis and the promotion of fatty acid oxidation by AMPK activation. We used the HFD-induced NAFLD animal model for demonstrating the therapeutic effect of capsaicin from the mesenteric adipose tissue.

## Materials and methods

### Animal and capsaicin treatment

Male C57/BL6 mice (8 weeks of age) were purchased from Orient Bio Inc. (Gyeonggi, Korea). The mice (*n *=* *7 per each group) were fed either 60% kcal HFD (Research Diets Inc., New Brunswick, NJ, USA) or methionine choline-deficient (MCD) (ICNBiomedicals, Costa Mesa, CA, USA) diet for 8 weeks. For tropical applications, 0.075% capsaicin was mixed with hydrophilic cream base (Sigma, St, Louis, MO, USA). HFD-induced mice (HFD mice) were treated daily with 100 mg capsaicin cream onto shaved abdominal skin for 8 weeks. Hepatic fat accumulation was evaluated by measuring liver weight and Oil Red O staining. The expressions of lipid metabolism factors were analyzed by reverse transcription-polymerase chain reaction (RT-PCR) and western blot analysis.

All the experiments were performed in compliance with the guidelines of the University of Ulsan Animal Care and Use Committee and conformed to the guidelines of the National Institutes of Health.

### Histological analysis

Liver specimens were fixed overnight in buffered formaldehyde (10%) and embedded in paraffin. To evaluate the accumulation of liver fat, hematoxyline and eosin stain were performed. For oil-red staining, the liver tissues were immediately frozen in liquid nitrogen and fixed in Tissue-TeK OCT compound (Sakura Finetek USA, Torrence, CA, USA). Sections were stained with 0.5% Oil Red O solution (Sigma) for 10 min at room temperature. The sections were stained with hematoxylin to visualize the nucleus and then washed with distilled water and mounted using a water-based mounting medium.

### Plasma biochemical assay

Blood samples were collected from the heart in heparinized tubes, and the plasma was isolated immediately by centrifugation (3000 rpm, 4°C, 15 min). Plasma transaminase (ALT and AST) and triglyceride (TC and TG) were determined by EnzyChrom^TM^ Aspartate Transaminase assay kit, EnzyChrom^TM^ Alanine Transaminase Assay Kit, EnzyChrom^TM^ Triglyceride Assay Kit, and EnzyChrom^TM^ Cholesterol Assay Kit (BioAssay Systems, Hayward, CA, USA).

### RNA extraction and RT-PCR

The collected liver tissues were homogenized with TRI reagent (Invitrogen, Carlsbad, CA, USA). Total RNA was reverse-transcribed using an oligo dT (Invitrogen) and M-MLV reverse transcription kit (Promega, Madison, WI, USA). PCR was performed with Go Taq DNA polymerase (Promega) under the following conditions: initial denaturation at 94°C for 2 min, cycling at 94°C for 30 s, at appropriate annealing temperature for 30 s and 72°C for 30 s, with a final extension at 72°C for 5 min. Specific primer sequences were used in this study shown in Table 1. PCR product was electrophoresed on 1.5% agarose gel and stained with SYBR safe DNA Gel stain dye (Thermo Fisher Scientific, Waltham, MA, USA). Band intensity was determined by Image J software 1.42v (NIH, USA) and normalized to GAPDH.

**Table 1.** Gene-specific primer sequences used in PCR amplification.

Gene	primer sequence (5′→3′)
ACC	F : AGGAGGACCGCATTTATCGAC
	R : TGACCGTGGGCACAAAGTT
CPT1a	F : GAGTTTAGGGTGAGCCTGAA
	R : GGACTAAAGGTGTGCACTAA
CD36	F : AATATAACTCAGGACCCCGA
	R : TAGGCTGATTGGGTCATCTA
FAS	F : CTGCGGAAACTTCAGGAAATG
	R : GGTTCGGAATGCTATCCAGG
G6Pase	F : TGATAGGATGGGGATGGAAT
	R : ATTCCCAACACTCTGGAATC
PEPCK	F : CTTCTCTGCCAAGGTCATCC
	R : AGTGAGAGCCAGCCAACAGT
GAPDH	F : AGGTCGGTGTGAACGGATTT
	R : GGGGTCGTTGATGGCAACA

### Western blot analysis

Tissues were homogenized with 1× passive lysis buffer (Promega) supplemented with protease inhibitor cocktail (Sigma) and Xpert Phosphatase Inhibitor Cocktail Solution (GenDEPOT, Barker, TX, USA). Lysates were quantified using BCA protein assay kit (Thermo Fisher Scientific) and an equal amount of protein was electrophoresed on 8–10% SDS-polyacrylamide gel and transferred onto polyvinylidene fluoride membranes. Blots were blocked in 1× TBS-tween 20 containing 5% non-fat milk (Difco, Sparks, MD, USA) and incubated with primary antibodies. Antibodies including AMPK, p-AMPK, ACC, FAS, CPT1a, PEPCK-C, G6Pase, CD36, and PPAR-α were purchased from Cell signaling and α-tubulin, GAPDH were purchased from Santa Cruz Biotechnology (Dallas, TX, USA). Next day, the membranes were washed with 1× TBS-T and reacted with horseradish peroxidase-conjugated secondary antibodies (1:10,000) (Santa Cruz Biotechnology) and visualized by SuperSignal^TM^ West Pico Chemiluminescent substrate (ThermoFisher Scientific). Band intensity was measured by Image J software 1.48v (NIH, USA) and was normalized to the α-tubulin.

## Results

**Figure 1.**
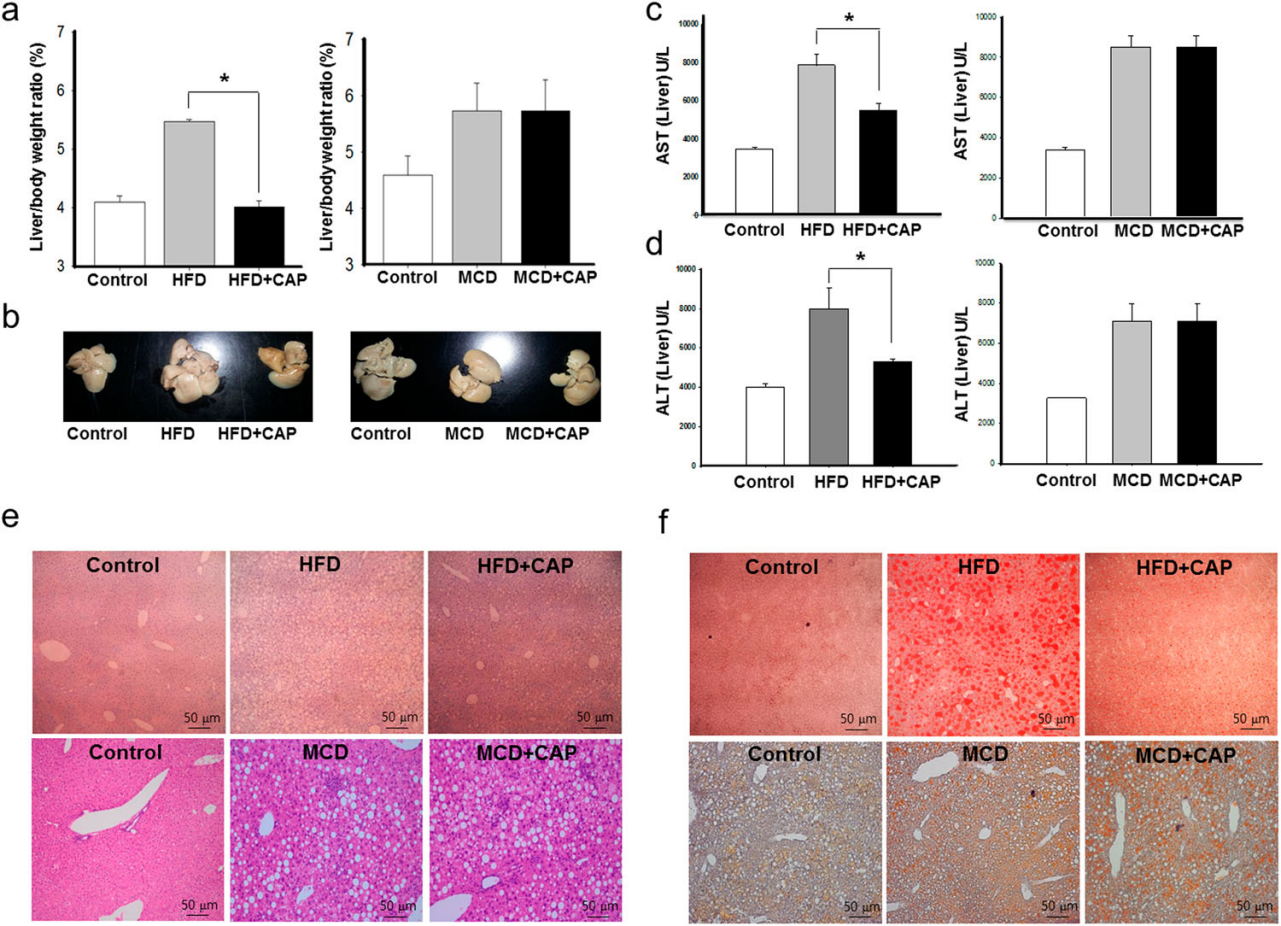
Topical application of capsaicin decrease hepatic fat accumulation in the HFD-induced obese mice. Mice fed an HFDcontinuously for 14 weeks. After 7 weeks the mice were divided into two groups, one of which received treatment with 0.075% capsaicin cream for the remaining 7 weeks, the other control cream. Control group of mice fed a general diet continuously for 14 weeks. (a) Liver/body weight ratio, (b) Representative anatomical photographs of liver, (c) AST, (d) ALT, (e) Representative histological images of liver tissues (H&E staining), (f) Representative histological images of liver tissues (Oil-red O staining). Body and tissue weight were measured with an analytical balance. Data are mean ± s.e.m. *P <* *.01.

Given that topical application of capsaicin limited fat accumulation in adipose tissues in our previous study (Lee et al. 2013), our initial study here examined whether capsaicin treatment alleviates hepatic fat accumulation in obese mice. We measured the ratio of liver weight to the body weight of mice after 8 weeks of capsaicin application. While the ratio of liver weight to body weight was found to be increased in HFD mice, the capsaicin treatment prevented this increase in the HFD mice (Figure 1(a) and (b)). There was no difference in food intake of HFD between capsaicin treatment and control (Suppl Fig. 1). The AST and ALT values, which were elevated in HFD mice, were recovered by capsaicin treatment (Figure 1(c) and (d)). Cross-sections of liver tissue and oil-red staining also showed a marked decrease of lipid droplets in the capsaicin-treated HFD mice (Figure 1(e) and (f)). The standard HFD provokes obesity, metabolic syndrome, and hepatic steatosis (Asai et al. 2014). Compared to lean, insulin-sensitive MCD diet-fed mice, which developed severe NASH, obese, the insulin-resistant ‘Western diet’-fed mice exhibited only mild NASH (Machado and Cortez-Pinto 2014).

**Figure 2.**
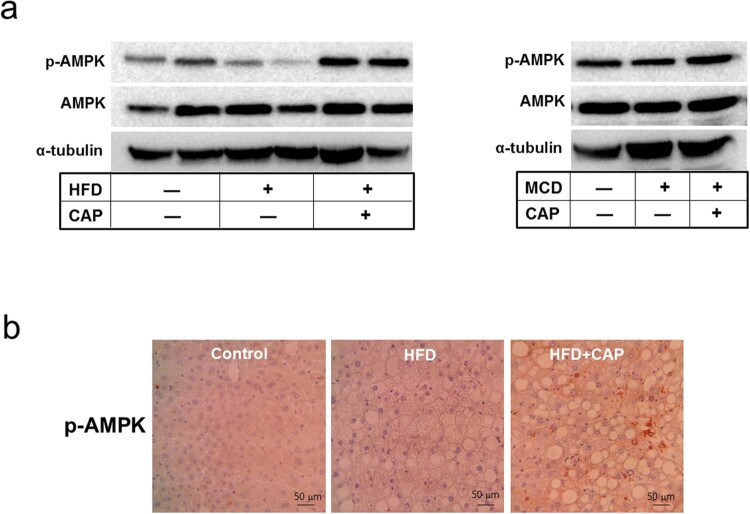
Capsaicin activates phosphorylation of AMPK in liver of the HFD-induced obese mice. (a) Western blot analysis of AMPK protein in the liver, (b) Immunolabeling of phosphor-AMPK in liver of the HFD-fed mice.

To examine the molecular mechanism underlying capsaicin’s inhibition of fatty liver from HFD-induced obese mice, we addressed whether the topical application of capsaicin activates AMPK in liver. Hepatic AMPK in the HFD mice was less phosphorylated than lean mice, whereas phosphor-AMPK level was reversely increased in the capsaicin-treated mice (Figure 2). This result suggests that the AMPK-relevant pathway is associated with capsaicin effect on the fatty liver of HFD model.

**Figure 3.**
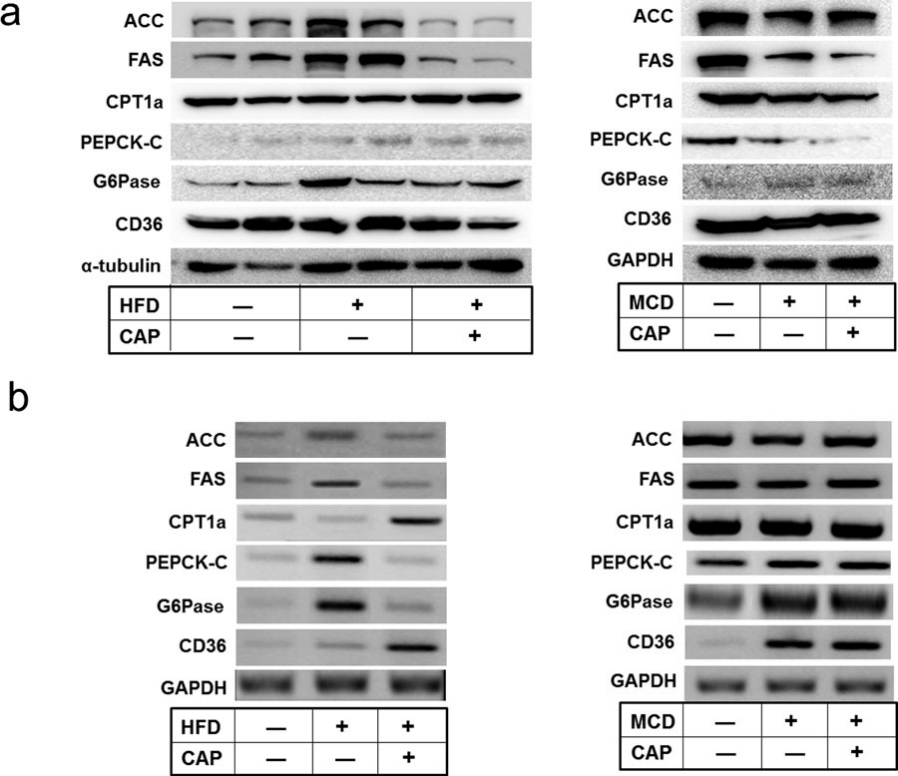
Capsaicin regulates the fatty acid metabolism-related genes in liver of HFD-induced obese mice. (a) Western blot analysis, (b) RT-PCR analysis of ACC, FAS, CPT1a, PEPCK-C, G6Pase, and CD36 in liver of the mice fed either with HFD or MCD diet.

Activation of hepatic AMPK leads to increased fatty acid oxidation with simultaneous inhibition of hepatic lipogenesis and gluconeogenesis (Hardie 2004). To connect activation of AMPK in liver and the topical application of capsaicin, we analyzed expression of the lipid metabolism-associated enzymes in the AMPK signaling pathway. The protein and mRNA levels of lipogenesis enzymes, such as fatty acid synthetase (FAS) and acetyl-CoA carboxylase (ACC) were found to be reduced by topical application of capsaicin whereas *β*–oxidation-associated enzyme, such as carnitine palmitoyltransferase1 (CPT1) was found to be increased (Figure 3). The mRNA level of fatty acid translocase (CD36), which promotes free fatty acids influx, was also increased by capsaicin treatment. In addition to inhibiting ACC and decreasing intracellular lipid stores, AMPK lowers the expression of liver glucose-6-phosphatase (G6Pase) and phosphoenolpyruvate carboxykinase (PEPCK)-C, which are involved in gluconeogenesis. In our study, capsaicin treatment reduced significantly the mRNA levels of PEPCK-C and G6Pase, indicating that capsaicin application suppresses lipogenesis but promotes fatty acid oxidation in the liver of HFD obese mice via the AMPK signaling pathway.

**Figure 4.**
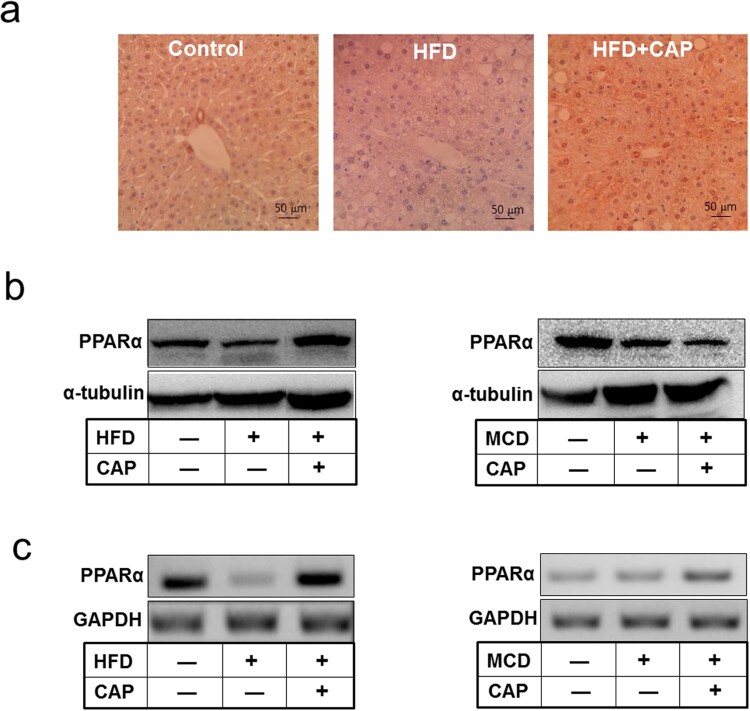
Capsaicin promotes adiponectin signaling in liver of the HFD-fed mice. (a) Immunohistochemical staining of adiponectin in liver tissue, (b) Western blot analysis, (c) RT-PCR analysis of PPAR-α.

Adiponectin increased fatty-acid combustion and energy consumption, which led to decreased triglyceride content in the liver through activation of AMPK and PPAR-*α* pathways (Polyzos et al. 2010). Given these established roles of adiponectin on NAFLD, we investigated whether adiponectin is involved in less hepatic fat accumulation by topical application of capsaicin. Immunohistochemical staining showed that adiponectin binding to liver tissues was increased in HFD-fed mice with capsaicin treatment compared to that of HFD mice without capsaicin treatment (Figure 4(a)). Binding of plasma adiponectin to adiponectin receptor 2 (AdipoR2) on liver membrane activates PPAR-*α* pathways. We observed that the capsaicin treatment increased phosphorylation of AMPK and level of PPAR-*α* protein in HFD-induced obese mice (Figure 4(b) and (c)). These data suggest that lower fatty liver symptoms by topical application of capsaicin are induced by AMPK-mediated adiponectin signal.

## Discussion

HFDis a highly studied approach to develop NAFLD. Interestingly, we showed that HFD increased hepatic fat, but capsaicin treatment decreased it to the level of lean diet-fed mice. HFD-induced genes were involved in *de novo* lipogenesis and fatty acid combustion in liver (Yamauchi et al. 2003; Machado and Cortez-Pinto 2014). These findings suggest that the topical application of capsaicin may promote lipogenesis and inhibit *β*-oxidation in HFD-induced mice. On the basis of our data, it is likely that the effect of capsaicin is associated with adipose tissues and leads to less fat accumulation in the liver of the HFD-induced obese mice.

HFD induces obesity and impaired metabolic syndrome. Most of the adipose tissue-derived proteins are elevated in obesity and may contribute to systemic inflammation and liver damage. Altered adipokines production of the adipose tissues in obesity has been implicated in the pathophysiology of diverse diseases including NAFLD (Radin et al. 2009). In our previous study, we found that the topical application of capsaicin on HFD-induced obese mice increased adiponectin level in mesenchymal adipose tissues.^3^ Although adiponectin is highly abundant in human serum, its levels are reduced in obese patients and patients with hepatic steatosis of NASH. It is suggested that adiponectin could minimize fat accumulation in the liver (Awazawa et al. 2009). In the present study, we found that capsaicin treatment increased adiponectin level in the liver of HFD-fed obese mice. Adiponectin antagonizes excess lipid storage in the liver and prevents inflammation and fibrosis (Kadowaki et al. 2006). Since adiponectin secreted from mesenteric adipose tissues can act directly on hepatic tissues and produce beneficial effects on lipid metabolism, adiponectin seems to be involved in the capsaicin-induced inhibition of hepatic lipid accumulation. We sought to investigate whether adiponectin increase due to the topical application of capsaicin can reduce liver fat in the HFD-induced NAFLD animal models. Phosphorylation and activation of the AMPK stimulate adiponectin in the liver (Yamauchi et al. 2002). Capsaicin reduced inflammation in liver without changes in body weight and adiposity (Kang et al. 2011). The effect of capsaicin on this reduction was suggested by hepatic AMPK activation, systemic increases in the concentration of adiponectin and increased AdipoR2 in liver. Our study shows that the topical application of capsaicin resulted in AMPK activation, which might be stimulated by adiponectin in the liver. In addition, capsaicin treatment also stimulated CPT-1 and CD36 expression, which are associated with β-oxidation and fatty acids influx. The increased hepatic synthesis of fatty acid, and possibly their increased uptake, eventually reaches equilibration by an increased rate of mitochondrial β-oxidation and an increased rate of hepatic triacylglycerol secretion, allowing stabilization of the amount of fat in the liver. In contrast, capsaicin reduced molecules such as ACC, FAS, PEPCK-C, and G6Pase, which are involved in *de novo* lipogenesis and gluconeogenesis in the liver. AMPK activation results in the inhibition of ACC, the rate-limiting enzyme controlling fatty acid synthesis. The reduced expression of gluconeogenic enzymes such as PEPCK-C and G6Pase was associated with elevated phosphorylation of AMPK in adiponectin transgenic mice (Combs et al. 2004). Our data indicate that the topical application of capsaicin accounts for the inhibition of hepatic lipogenesis and endogenous glucose production.

The common causes of mild increase in AST and ALT levels include NAFLD, hepatitis C, and alcoholic fatty liver disease. AST and ALT are reasonably sensitive indicators of liver damage or injury from different types of diseases or conditions, although higher-than-normal levels of these liver enzymes should not be equated with liver disease (O'Shea et al. 2010). We showed that AST and ALT are recovered by capsaicin treatment in HFD mice. Although there is no proven pharmacotherapy for the treatment of NAFLD, therapeutic strategies to upregulate adiponectin levels, such as TZD administration are expected to be effective for the treatment of the metabolic syndrome including NAFLD (Iwaki et al. 2003; Sharabi et al. 2007). Recent study showed that the elevation of adiponectin level by natural product supplementation might benefit patients with metabolic syndrome and its animal model (Kessoku et al. 2016). The result of this study suggests that topical application of capsaicin could potentially be beneficial in preventing the complications of NAFLD.
